# Intramedullary Spinal Cord Cavernous Angiomas in Familial Cerebral Cavernous Malformations

**DOI:** 10.3390/genes17080845

**Published:** 2026-07-23

**Authors:** Marialuisa Zedde, Vincenzo D’Agostino, Francesca Romana Pezzella, Piergiorgio Lochner, Vincenzo Seneca, Giuseppe Catapano, Rosario Pascarella

**Affiliations:** 1 Neurology Unit, Stroke Unit, AUSL-IRCCS di Reggio Emilia, 42123 Reggio Emilia, Italy; 2 Neurology and Stroke Unit, Ospedale del Mare, ASL Napoli 1 Centro, 80147 Napoli, Italy; 3 Neuroradiology Unit, Ospedale del Mare, ASL Napoli 1 Centro, 80147 Napoli, Italy; 4 Neurology Unit, Ospedale Santa Maria Goretti, 04100 Latina, Italy; 5 Department of Neurology, Saarland University Medical Center, University of Saarland, Kirrberger Street 100, D-66421 Homburg, Germany; 6 Neurosurgery Unit, Ospedale del Mare, ASL Napoli 1 Centro, 80147 Napoli, Italy; 7 Neuroradiology Unit, Ospedale Santa Maria della Misericordia di Rovigo, AULSS5 Polesana, 45100 Rovigo, Italy

**Keywords:** cavernous angioma, spinal cord, familial cavernous malformation, FCCM, CCM, SCCM, MRI

## Abstract

Background: Spinal cord cavernous malformations (SCCMs) are vascular malformations characterized by blood-filled cavities, often leading to neurological deficits. Familial cerebral cavernous malformation (FCCM) is a hereditary condition that predisposes individuals to develop multiple cavernous angiomas. Understanding the incidence, clinical presentation, and management strategies for spinal cavernous angiomas in the context of FCCM is crucial for improving patient outcomes. Methods: This narrative review synthesizes existing literature on SCCMs in patients with FCCM. A comprehensive search was conducted across multiple databases, including PubMed, Scopus, and Web of Science, utilizing keywords such as “spinal cavernous angiomas” and “familial cerebral cavernomatosis”. In addition, a further search was performed among papers discussing FCCM and SCCM, respectively. Discussion: In the paucity of published data about the presence of SCCMs in FCCM, the review highlights the clinical manifestations of SCCMs in both sporadic disease and FCCM, including recurrent hemorrhagic episodes and progressive neurological deficits. Although mutations in the *KRIT1*, *CCM2*, and *PDCD10* genes play a significant role in the pathogenesis of FCCM, no single gene mutation has been identified as predisposing to SCCMs. Diagnosis was usually reached in patients symptomatic for spinal cord bleeding. Surgical intervention remains the primary treatment modality; however, the decision-making process is complicated by the potential for new lesion development. Conclusions: SCCMs in FCCM present distinct challenges in diagnosis and management and their prevalence is probably underestimated. This review underscores the need for heightened awareness among clinicians regarding the hereditary nature of these lesions. Future research should focus on the molecular mechanisms underlying FCCM, aiming to develop targeted therapies and improve clinical outcomes for affected individuals.

## 1. Introduction

Cerebral cavernous malformations (CCMs) are prevalent vascular anomalies within the central nervous system (CNS), affecting an estimated 0.16–0.40% of the worldwide population [1,2]. The distinctive characteristics of CCMs include thinned, enlarged, and abnormally permeable plasma membranes in endothelial cells, which heightens the risk of complications such as strokes, seizures, and focal neurological deficits [3]. At present, there are no known effective pharmacological interventions for these disorders [4].

While CCMs rank as the second most frequent type of vascular malformation in the CNS, trailing only developmental venous anomalies (DVAs), less information is available regarding their spinal equivalent, spinal cavernous malformations (SCCMs) [5]. SCCMs are defined as well-circumscribed aggregates of enlarged blood vessels that do not involve the surrounding brain parenchyma [6]. These malformations are not detectable through catheter angiography, earning them the designation of angiographically silent. Various terms, including cavernous angioma, cavernoma, cavernous hemangioma, and cavernous venous malformation, have been applied to these lesions, with prevalence estimates varying between 0.1% and 0.5% [7,8].

CCMs are composed of abnormally structured slow-flow capillaries that create multiple mulberry-like distensions, lacking normal intervening brain architecture [4,9]. These individual cavernomas are frequently encircled by hemosiderin, a marker of past bleeding from the abnormal capillaries [10]. Most cases of sporadic CCM present as single lesions, sometimes in conjunction with DVAs. In contrast, familial or hereditary CCM (FCCM), which arises from an autosomal dominant genetic mutation, typically results in multiple lesions, mainly located in the supratentorial region rather than in the infratentorial area [5]. Although CCMs can be detected in infants and children, they most commonly present between the second and fifth decades of life, often discovered incidentally or alongside symptoms such as seizures, focal neurological deficits, headaches, or cerebral hemorrhage. Skin and retinal vascular lesions are observed in approximately 9% and 5% of affected individuals, respectively, with around 50% of those with FCCM remaining asymptomatic throughout their lives [11].

The main features of FCCM is the presence of multiple CCMs in the brain and spinal cord. Although sporadic CCMs can appear in the spinal cord, research has primarily concentrated on occurrences in the brain. The number of CCMs can increase over time, and individual lesions can vary significantly in size. The individual CCMs burden can range from 1–2 to more than 100, being the average number of 6–20 lesions. FCCMs is an autosomal dominant disease, resulting from germline loss-of-function mutations in the *CCM1* (*KRIT1*), *CCM2* (*MGC4607*), and *CCM3* (*PDCD10*) genes [12]. There is currently no established consensus on clinical diagnostic criteria for FCCM. In cases where genetic testing is not available, the presence of both CMs and DVAs can help in differentiating between sporadic and familial forms. In individuals without a history of radiation exposure, a solitary cavernous malformation with an associated DVA typically suggests a sporadic origin. Conversely, the presence of multiple CCMs without a DVA suggests FCCM. In fact, patients with a single CCM with an associated DVA and no family history are highly likely to have sporadic disease, and this information can be used in low-resource settings to avoid expensive genetic testing [10]. DVAs are a highly prevalent incidental finding (roughly 3% of the population) [13] without significant hemorrhagic risk. However, DVAs may contribute to the formation of adjacent CCMs, occurring in up to 7% of patients over their lifetime [14]. Current guidelines suggest genetic testing in patients with multiple CCMs (particularly without DVA and history of brain irradiation) and/or positive family history [15].

CCMs are not only intra-axial lesions, but they may also arise in cranial nerves, ventricles, cavernous sinuses, and dural sinuses, as well as in other structures, such as the tentorium, falx, retina, and orbit [16,17,18,19,20,21,22,23,24,25]. In the spine, apart from the classic intramedullary malformations, these lesions can be found in the epidural space and intradural extramedullary areas [22,23,24,25,26,27]. Spinal cord cavernous malformations (SCCMs) are uncommon, well-defined intramedullary vascular lesions characterized by a mulberry-like morphology, similarly to the brain-located lesions. Histologically, SCCMs are made of multi-lobulated, sinusoidal spaces lined by endothelium, lacking tight junctions and neural parenchyma. While they share histological similarities with CCMs, calcification is observed less frequently in SCCMs compared to their cranial equivalents [28,29].

A literature search was performed in order to define the already published data about SCCMs in patients with FCCM. We used Pubmed, Scopus and Web of Science to look for any association between FCCM and spinal cord CCM. Scopus and Web of Science did not add any papers to the search performed on Pubmed. We used the following key words in Pubmed: [((((cerebral cavernous malformation[MeSH Terms]) AND (familial cavernous malformation[MeSH Terms])) AND (cervical spinal cord[MeSH Terms])) AND (thoracic cord[MeSH Terms])) AND (cord, lumbar[MeSH Terms])]; only three papers were found [30,31,32]. Even using back and forward citations of these papers, we did not find relevant sources of added information. Then, we made a more general search with two different lines, one focused on FCCM and the other one focused on SCCMs. A systematic approach with the available data was not possible, so we described the output of our search in a narrative review based on the two above-mentioned lines.

Therefore, this narrative review aims to identify and elaborate on the limited existing evidence regarding the characteristics and management of SCCMs in patients with FCCM, thereby highlighting current knowledge deficiencies. The focus of this review is specifically on intramedullary SCCMs.

## 2. Starting from FCCM

Two seminal studies estimated the prevalence of CCMs to be 0.39% and 0.47%, based on comprehensive MRI assessments [33,34]. Subsequent research has validated these findings, with current estimates for the prevalence of CCMs in the general population ranging from 0.1% to 0.5% [7,8]. The Scottish Intracranial Vascular Malformation Study (SIVMS) reported an annual incidence rate of 0.56 per 100,000 among individuals aged 16 and older [35]. Further studies have indicated prevalence rates varying between 0.16% and 0.5% [2,9,36]. A more recent prospective population-based imaging study conducted by the Mayo Clinic in adults aged 50 to 89 years found a prevalence of 0.46%, with a mild sex imbalance (men 0.51% vs. women 0.41%) [9]. The estimated population prevalence of FCCM ranges from 1 in 5000 to 1 in 10,000, with a recent analysis of exome sequencing databases suggesting a prevalence of 1 in 3300 to 1 in 3800 individuals [37]. Notably, none of these studies specifically examined the prevalence of SCCMs.

The mean age for the presentation of these vascular malformations is approximately 37 years, although they can occur at any age [5]. The Mayo Clinic study noted that the prevalence of CCMs associated with DVAs tends to increase with age [14]. While detailed data on the racial or ethnic distribution among CCM patients is scarce, a notably higher prevalence of FCCM has been reported in Hispanic populations, particularly in the American Southwest, attributed to a common mutation in the CCM1 gene [38,39].

CCMs are generally detected through T2-weighted gradient-echo (GRE) or Susceptibility-Weighted Imaging (SWI) MRI sequences. Some studies indicate that as many as 75% of sporadic CCM cases may actually be familial, appearing as asymptomatic lesions that obscure an autosomal dominant inheritance pattern [40]. Currently, mutations are identified in 40% to 78% of CCM cases based on recent research, suggesting that molecular screening cannot definitively exclude the disease [41,42,43,44]. Variations in detection rates may arise from including patients with a history of unexplained cerebral hemorrhage. The Spanish Society of Neurology recommends genetic testing for CCM-related genes in patients diagnosed with stroke [45].

Historically, three genes have been associated with CCMs:

- *CCM1/KRIT1* located on chromosome 7 (7q21–q22);
- *CCM2/MGC4607* found on the short arm of chromosome 7 (7p13);
- *CCM3/PDCD10* located on chromosome 3 (3q26.1).

Several mutations (more than 200) have been documented in patients with FCCM, typically presenting each one with a low risk of recurrence. Some mutations are population-specific. In Spain and Portugal, a specific 14 bp deletion in exon 5 of the CCM2 gene is notably prevalent [46]. Conversely, the c.1363T>C mutation in CCM1 gene, common in Hispanic populations [39], is not present in Spanish patients [47]. Again, in a Spanish cohort, 56% of mutations were found in CCM1 gene, while 33% and 6% affected CCM2 and CCM3 genes, respectively [42]. However, a significant number of families remain without identified mutations, with large deletions detected across all three CCM genes [43,48,49,50]. These genes encode scaffold proteins involved in angiogenesis and vasculogenesis, forming a ternary complex, and mutations that lead to loss of protein function are considered pathogenic. According with the type of mutation, patients with CCMs may present nonsense, frameshift, splice site, missense, silent mutations, and large deletions. Nonsense mutations introduce premature stop codons [e.g., c.902C>G (p.S301X) in *CCM1* gene], frameshift mutations are provoked by insertions or deletions [e.g., c.968_971dupCACC (p.Ile325ThrfsX11) in *CCM1* gene], and splice site mutations may affect invariant or consensus sequences, all resulting in truncated proteins. Conversely, missense mutations involve a change in a single amino acid, while silent mutations do not affect protein function, being the main source of variants of uncertain significance. Frameshift mutations are the most common pathogenic variants [42,43]. The introduction of multiplex ligation-dependent probe amplification (MLPA) has facilitated the detection of deletions across multiple exons, which are not easily identified through direct sequencing. Recently, quantitative multiplex PCR of short fluorescent fragments has also been employed to detect deletions [43]. Nearly 40 deletions have been documented in CCM genes [43,48,49,50,51,52,53,54,55,56,57,58], and these should be considered pathogenic due to their likelihood of resulting in incomplete transcription.

Lastly, some polymorphisms may manifest during sequencing, both in exons and introns. Certain polymorphisms have been associated with an increased risk of CCMs, while others may predispose patients to debilitating symptoms rather than life-threatening conditions [53].

The pathogenesis of FCCM is outside the scope of this paper and is summarized in Table 1 [59].

Although CCMs were once considered congenital, the majority are now recognized as sporadic, typically identified in patients during their third and fourth decades of life. Approximately 85% of cases are classified as sporadic, with de novo formation frequently reported [3,9]. Recent studies have identified somatic mutations in *MAP3K3* and *PIK3CA* as prevalent in sporadic CCMs and SCCMs [60,61,62,63,64]. However, the role of *MAP3K3* mutations as primary contributors to the formation and pathogenesis of CCMs and SCCMs remains uncertain.

The etiology of CCMs associated with mutations in *CCM1*, *CCM2*, and *CCM3* has been described as a two-hit mechanism, involving mutations in two of the three CCM genes [59,65]. Recently, a three-hit model has been proposed for severe CCMs cases, suggesting that gain-of-function (GOF) mutations in the oncogene *PIK3CA*, combined with loss-of-function (LOF) mutations in *CCM1*, *CCM2*, or *CCM3*, may intensify the severity of the malformations [64]. Surprisingly, neither LOF mutations in *CCM1*, *CCM2*, or *CCM3* nor GOF mutations in *PIK3CA* alone have been shown to drive CCMs in adult mice [64]. Ren et al. [66] reported that 66% of patients with the *MAP3K3* I441M mutation have not any additional known mutations in CCM genes (*CCM1, CCM2, CCM3, PIK3CA*). They proposed a mouse model where the *MAP3K3* I441M mutation is exclusively expressed in CNS endothelial cells, resulting in the development of CCMs and SCCMs similar to human pathology [66]. In vivo time-lapse imaging of the mature brains of *MAP3K3* I441M mice revealed the formation of CCMs characterized by dilated, low-flow, and leaky caverns. The expression of *MAP3K3* I441M initiated CCM development by upregulating cytoskeletal production and increasing the size of individual endothelial cells. Furthermore, the study demonstrated that *MAP3K3* I441M can activate the mTOR (mammalian target of rapamycin) pathway, with rapamycin treatment shown to slow CCM progression [66].

Only isolated cases of “silent” SCCMs have been reported in patients with FCCMs, though routine spinal cord screening is not conducted in these individuals [32]. Notably, a recent ongoing prospective study examining the natural history of FCCM from both clinical and neuroradiological perspectives has not incorporated spinal cord imaging into its evaluations [67].

## 3. Starting from SCCMs

SCCMs account for approximately 5% of all cavernous malformations within CNS and 5–12% of intraspinal vascular malformations [38,39,40,41,42]. Both rarity and lack of investigations in asymptomatic patients contribute to the missed information about the true prevalence. Neuroimaging investigations, mainly performed in patients with spinal bleeding due to SCCMs, indicate that about 40–47% of patients also have a CCM, while approximately 34% have an associated DVA; 12% of patients have FCCM [68]. Notably, multiple SCCMs without extensive cranial involvement have been infrequently reported [69,70,71]. However, no specific genetic mutations predisposing individuals specifically to SCCMs have been identified [72]. Although SCCMs can occur in familial contexts, most documented cases appear sporadic, lacking a unique genetic signature associated with these lesions [73].

The clinical presentation of intra-axial SCCMs depends on the eloquence of the surrounding tissue, affected by bleeding. It is unlikely that a hemorrhage due to an intramedullary SCCM is asymptomatic, but asymptomatic patients rarely underwent spinal cord MRI [28,29,74,75,76,77]. They occur at an estimated rate of approximately 1.86 per 100,000 individuals [76]. The average age at presentation is 39.1 years (range 2–80 years) with a slight male predominance (1.1:1.0) [78]. Age and gender are not effective indicators for identifying patients with this condition. Histopathologically, SCCMs are characterized by an abnormally complex vascular network of varying sizes, without involvement of large arteries or veins [79]. Clinical manifestations of SCCMs are related with single or repeated spinal cord hemorrhages [79,80].

As previously said, the decision to investigate for spinal lesions has been usually driven by neurological examinations, with most reported cases involving symptomatic patients. SCCMs are distributed throughout the spinal column, roughly correlating with the volume of each spinal segment. The majority of lesions are found in the thoracic (55%) or cervical (38%) regions, followed by the cervicothoracic (2.4%) or thoracolumbar (0.6%) junctions; only 2.1% are in the lumbar region, and 1.7% are found at the conus medullaris [76,78,81].

Significant research has focused on identifying genetic etiologies in patients with CCMs and SCCMs [33,82,83,84,85,86,87]. A genetic predisposition may account for multiple lesions in an individual, as 1 out of 7 patients with SCCMs also have CCMs [78,86,87]. The primary barrier to standardized imaging of the entire neuraxis in patients with cavernous malformations is the lack of indication for intervention in asymptomatic lesions.

The clinical pattern of intramedullary SCCMs diverges from that of hemispheric and brainstem malformations [27]. Overall, 50–70% of patients exhibit slow and progressive myelopathy, likely due to the spinal cord’s low tolerance for chronic local irritation associated with the malformation [88]. Relapsing and remitting neurological deficits are also common [88]. The annual hemorrhage rate for symptomatic intramedullary SCCMs appears to be slightly higher than for CCMs, estimated at 1.4–4.5% per year. Surgical resection of symptomatic lesions is generally recommended due to the critical nature of the surrounding tissue. Acute neurological deficits tend to respond more favorably to prompt surgical intervention, especially in patients with newly identified lesions.

Finally, as previously noted, up to 40% of patients with intramedullary SCCMs have associated CCMs, reinforcing the recommendation for neuraxis screening in newly diagnosed intramedullary SCCMs [89]. Among reported cases of SCCMs, 10–15% had a family history [33,82,83,84,85,86,87]. These findings suggest that family history can be a valuable aspect of the assessment for patients presenting with neurological deficits, although the majority of cavernous malformations occur in individuals without a familial history.

## 4. Natural History

SCCMs are considered uncommon, with most available research consisting of retrospective case studies [90,91]. Initial studies suggested that surgical removal was both safe and effective, potentially leading to a selection bias that overlooks patients who are monitored instead of surgically treated. In a meta-analysis focused on intramedullary SCCMs, only 10.1% (64 out of 631 patients) did not receive surgical intervention [78]. Thus, our comprehension of the natural progression of SCCMs relies on a limited number of non-operative cases and the preoperative experiences of those who underwent surgery.

This diagnostic pathway may indicate a bias where patients represented in surgical series frequently exhibit symptoms. While over 99% of reported instances are symptomatic, it is important to acknowledge that cavernous malformations can also occur incidentally and remain asymptomatic [69,78,92]. Patients with symptoms generally show deficits corresponding to the malformation’s anatomical position, with around 60.5% presenting motor deficits, 57.8% sensory deficits, 33.8% experiencing pain, and 23.6% having bowel and/or bladder dysfunction; 0.5% might also face respiratory issues [78]. Several efforts have been made to classify the presentation of patients with spinal cavernous malformations into five distinct groups: (1) discrete episodes of neurological decline with varying recovery; (2) gradual, progressive neurological deterioration; (3) acute onset with rapid decline; (4) sudden onset of mild symptoms progressing to gradual deterioration; and (5) immediate onset of back pain without neurological symptoms [77,93]. Although these classifications can aid in discussions about patient expectations, no specific lesion characteristics have been identified to reliably predict patient categorization.

A meta-analysis conducted by Badhiwala et al. in 2014 [78], which aggregated data from 40 case series involving 632 patients, found that 45.4% exhibited acute or stepwise symptoms, while 54.6% showed progressive deficits. The two largest published series presented differing trends: Liang et al. [94] reported that 73 out of 96 patients (76%) experienced progressive decline, whereas Mitha et al. [77] noted that 50 out of 80 patients (63%) presented with acute symptoms. In a more recent investigation of 48 cases, Reitz et al. [81] observed that only 18.8% experienced slow, progressive decline, while the rest exhibited sudden acute episodes, followed by either recurrent acute deteriorations (33.3%) or gradual decline (41.7%). The reasons behind these varied presentations and inconsistent statistically significant patterns across studies remain unclear.

Most of the existing literature focuses on patients who have undergone surgical procedures or have been referred to neurosurgeons, leading to nearly 90% of reported outcomes being tied to surgical interventions [78]. This introduces a selection bias that may distort results toward more severe cases. Conversely, hemorrhage rates might be underestimated, as only symptomatic bleeds are usually clinically identified. Most hemorrhage rate estimates assume that cavernous malformations are congenital lesions. The documented annual hemorrhage rate for spinal cavernous malformations is reported to be between 1.4% and 6.8%, with Badhiwala et al. [78] indicating a rate of 2.1% (95% confidence interval (CI) 1.3–3.3%) [88,95,96,97,98,99,100]. While discussing hemorrhage rates with patients is important, the unpredictable neurological progression in individual cases complicates this understanding. Furthermore, there is evidence supporting the spontaneous formation of cavernous malformations, as well as their development in patients after radiation or in those with familial disease forms [33,76,82,83,84,85,86,87,101,102,103,104,105,106,107,108,109]. Assuming all lesions are congenital might lead to an underestimation of the hemorrhage rate.

Given the difficulties in estimating hemorrhage rates, many studies have concentrated on neurological outcomes for patients with SCCMs. Reitz et al. [81] assessed 48 cases that underwent surgery, with an average follow-up of 79.3 ± 35.2 months. They found that 70.8% maintained stable neurological function, 22.9% showed improvement, and 6.3% experienced deterioration post-surgery. They defined an unfavorable outcome as an American Spinal Injury Association (ASIA) grade of C or worse, indicating insufficient muscle strength below the level of injury. Their analysis revealed significant associations between adverse outcomes and factors such as thoracolumbar location, low preoperative Epstein and Cooper lower extremity grades, and preoperative ASIA grades A–C [110].

Prior to the findings reported by Reitz et al., Badhiwala et al. [78] analyzed a cohort of 24 patients and conducted a meta-analysis encompassing 40 studies on SCCMs that included 632 patients. Their main focus was on changes in neurological function from the initial presentation to the final follow-up. They identified a significant relationship between improved neurological outcomes and surgical resection performed within three months of symptom onset, with an odds ratio (OR) of 2.11 (95% CI, 1.31–3.41, *p* = 0.0002). Additionally, they found connections between neurological improvement and surgical hemilaminectomy (OR 3.20, 95% CI 1.16–8.86, *p* = 0.02), gross total resection (OR 3.61, 95% CI 1.24–10.52, *p* = 0.02), presentation with motor symptoms (OR 1.76, 95% CI 1.08–2.86, *p* = 0.04), and acute symptom onset (OR 1.72, 95% CI 1.10–2.68, *p* = 0.02). Notably, they found no correlation with factors such as age, gender, spinal level, lesion size, presence of CCMs, or family history of cavernomatosis [78].

The strength of a meta-analysis lies in its ability to assemble a sufficiently large cohort for statistically significant findings, even with methodological variations across studies. Most outcome data pertains to patients who have undergone surgical procedures, as 90% of individuals in published series received surgical interventions [78]. In this meta-analysis, only 10.1% of patients were non-operative. They reported that neurological function improved in 30.2% of non-operative patients compared to 51.5% in the surgical group. Stability of neurological function was seen in 58.5% of non-operative patients versus 37.8% in surgical patients, while deterioration occurred in 11.3% of non-operative cases compared to 10.7% in surgical cases. Traditionally, deeper lesions have been treated conservatively; however, for those surgically resected, the lesion’s location and depth did not significantly influence neurological outcomes.

These findings can be interpreted in several ways. One viewpoint is that surgical intervention enhances the rate of neurological improvement, while another highlights that 88.7% of patients remained stable or improved without surgery compared to 89.3% who underwent surgical intervention. A further interpretation suggests that similar proportions of patients experienced deterioration, regardless of whether they had surgery. However, limitations are present in these data. Since the studies are retrospective and lack randomization, it is plausible that the conservatively managed group had characteristics that contributed to poorer neurological outcomes or a lack of improvement. Furthermore, the meta-analysis indicated that a clinical history of less than five years was associated with better outcomes (OR 10.0, 95% CI 1.07–93.44, *p* = 0.04) [78], suggesting that longer follow-up may reveal different long-term outcomes for the non-operative cohort.

The annual hemorrhage rates for SCCMs are estimated to range from approximately 1.7% to 5.5% [110]. Badhiwala et al. [78] reported an annual bleeding rate of 2.1% based on their meta-analysis of 632 patients. For symptomatic individuals, the annual risk of hemorrhage is noted to be between 9.5% and 17.6% [85,111,112], while asymptomatic patients face a much lower annual risk of 0.8% [113]. Other studies have indicated bleeding rates for asymptomatic lesions of 1.4% and 1.6% per year [113,114]. A recent systematic review of outcomes for intramedullary SCCMs did not take into account the potential impact of familial CCM [115]. This review highlighted that an exceptionally high percentage of patients underwent surgical treatment, with 1005 out of 1091 patients (92.1%) receiving surgical resection, leaving only 86 patients (7.9%) being treated conservatively. Gross total resection was successfully achieved in 95.7% of cases, while partial resection occurred in 4.3%. The majority of lesions (60.2%) were located in the thoracic spine, with patients presenting primarily with motor deficits (60.4%) and sensory deficits (59.7%). Long-term follow-up indicated that surgical intervention improved neurological status in 36.9% of patients, remained stable in 55.8%, and deteriorated in 7.3% compared to their preoperative condition. In the conservative group, outcomes showed that 21.7% improved, 69.6% remained stable, and 8.7% experienced deterioration. Favorable long-term outcomes were associated with solitary lesions, a duration of preoperative symptoms of less than three months, and improved postoperative neurological status.

In a recent study involving 28 patients with intramedullary SCCMs (median age 47 years [IQR 36–61], 32% female), it was found that nine patients (32%) initially presented with symptomatic hemorrhage, ten (36%) with focal neurological deficits, and nine (32%) incidentally. During a median follow-up of 6.4 years (IQR 4.0–10.6), ten patients (36%) developed new symptoms, and five (18%) underwent surgical intervention. The annual rate of symptom occurrence was calculated at 5.1% (95% CI 2.5–9.4%). At final follow-up, 16 out of 26 patients (57%) were functionally independent (modified Rankin Scale ≤ 2) [116].

Overall, intramedullary SCCMs are regarded as more aggressive than their intracranial counterpart, given that the spinal canal has a lower tolerance for space-occupying lesions [78]. Pediatric SCCMs are considerably rarer, comprising only 1% of all intramedullary lesions [117]. Pediatric cases do not entirely mirror those in adults. While the natural history, surgical management, and long-term outcomes of adult SCCMs have been extensively documented in recent years—particularly in several large studies [28,77,78,81,94,118,119,120]—pediatric SCCMs are primarily represented in smaller case series or case reports [74,89,117,121,122,123,124,125,126,127,128,129,130,131,132]. A recent retrospective study evaluated the long-term natural history of SCCMs in children, reviewing a series of 20 pediatric patients (under 18 years old) from a total of 254 patients assessed at a single institution. They found that the annual hemorrhage rate in pediatric patients was 8.2% per patient per year, and following initial hemorrhagic events, the annual rate of recurrent bleeding increased to 30.7% per patient per year. In contrast, in 234 adult patients, the respective rates were 2.8% and 7.4%. Lesions located at the thoracic or lumbar levels (*p* = 0.002, OR = 3.425, 95% CI = 1.588–7.387) and recurrent hemorrhagic events (*p* = 0.005, OR = 3.209, 95% CI = 1.415–7.279) were more likely to follow an aggressive course. There were no significant differences in sex distribution, lesion location and size, types of symptoms, likelihood of severe neurological status, or immediate and long-term postoperative outcomes between pediatric and adult patients with SCCMs.

## 5. Neuroimaging

Neuroimaging methodologies for detecting CCMs have been standardized for the brain, yet these protocols are not consistently applied to the spinal cord. Diagnosing CCMs in the brain is often more complex than other vascular conditions, as these lesions are not visible through cerebral angiography. This method can only identify abnormal venous flow related to CCMs and any associated DVAs. Additionally, small and non-calcified lesions may not be captured on computed tomography (CT) scans [15]. Therefore, magnetic resonance imaging (MRI) is the preferred technique for evaluating CCMs. Ideally, MRI protocols should incorporate standard T1- and T2-weighted sequences, T2 FLAIR, and T2* sequences, along with susceptibility-weighted imaging (SWI) or similar susceptibility-sensitive techniques. Advanced imaging methods, including diffusion tensor imaging and task-based functional MRI, may also assist in surgical planning and navigation. Conventional T1- and T2-weighted MRI provides good sensitivity and specificity for detecting CCMs, making it the diagnostic method of choice [15,133]. Larger CCMs typically show a distinctive heterogeneous internal signal on conventional T1- and T2-weighted imaging, often referred to as a “popcorn appearance,” characterized by a hypointense rim due to hemosiderin from prior hemorrhages [133]. Based on their MRI characteristics and histopathology, CCM lesions are classified into four distinct types, which can help in predicting the risk of hemorrhage [72]. However, a similar classification for spinal cord cavernous angiomas has not yet been validated.

Research indicates that T2* gradient recalled echo MR imaging and SWI are more sensitive in detecting smaller CCMs compared to conventional T2 sequences [134]. Consequently, imaging should include susceptibility-sensitive sequences like SWI (Siemens) or comparable sequences such as Susceptibility Weighted Angiograms (General Electric), which are particularly adept at identifying and counting small (Type IV) CCMs and are regarded as the gold standard. A study by De Souza et al. found that SWI detected 1.7 times more lesions than T2* gradient recalled echo (GRE) sequences [135]. It is advisable that MRI for suspected CCMs in the brain and/or spinal cord incorporates SWI to confirm the presence of the lesion and assess for associated DVAs [15]. The association of a solitary CCM with a DVA increases the chances of a sporadic CCM diagnosis, although familial cavernous malformations can sometimes be linked with DVAs as well [10]. Cavernous malformations generally exhibit low-pressure hemorrhages and have characteristic channels, resulting in a relatively predictable appearance on MRI. Zabramski et al. [72] classified various types of lesions based on imaging and pathological features, emphasizing common MRI characteristics. T1-weighted, T2-weighted, and GRE sequences are particularly useful for non-invasive diagnosis. The heterogeneity of these lesions contributes to their classic “popcorn” or “mulberry” appearance. On T1-weighted images, lobulated masses appear isointense or hyperintense, with a hypointense edge indicating a reactive gliotic capsule [10,69,72,92,134,135,136,137,138]. On T2-weighted images, lesions predominantly display hyperintensity surrounded by a hypointense rim following hemorrhage [69]. In GRE images, cavernous malformations are generally hypointense, although larger lesions may contain a hyperintense core [69]. While GRE images are more frequently used for cerebral imaging, their effectiveness in diagnosing spinal lesions is limited.

Post-contrast studies typically show minimal to no enhancement, indicating the relatively low blood flow to these lesions. The average size of SCCMs is around 9.2 mm in maximum transverse diameter [78,81]. SCCMs are angiographically occult, and diagnosis relies significantly on MRI. They typically present as small (approximately 1 cm) intramedullary lesions with a hypointense rim and a reticulated core displaying heterogeneous signal intensity on T2-MRI. The Zabramski classification is commonly used to describe the MRI appearance of CCMs [72]. A modified version of this classification has been applied in recent studies to characterize SCCM appearances on MRI [133]. According to the modified classification, Type I lesions are generally hyperintense on T1-weighted images due to acute to subacute hemorrhage but display variable intensity on T2-weighted sequences. Type II lesions exhibit a mixed signal intensity on both T1- and T2-weighted images, reflecting hemorrhages of varying ages and a peripheral hypointense hemosiderin ring. Type III lesions represent chronic lesions that are iso- to hypointense on T1-weighted imaging and hypointense on T2-weighted imaging due to hemosiderin deposition.

**Figure 1:**
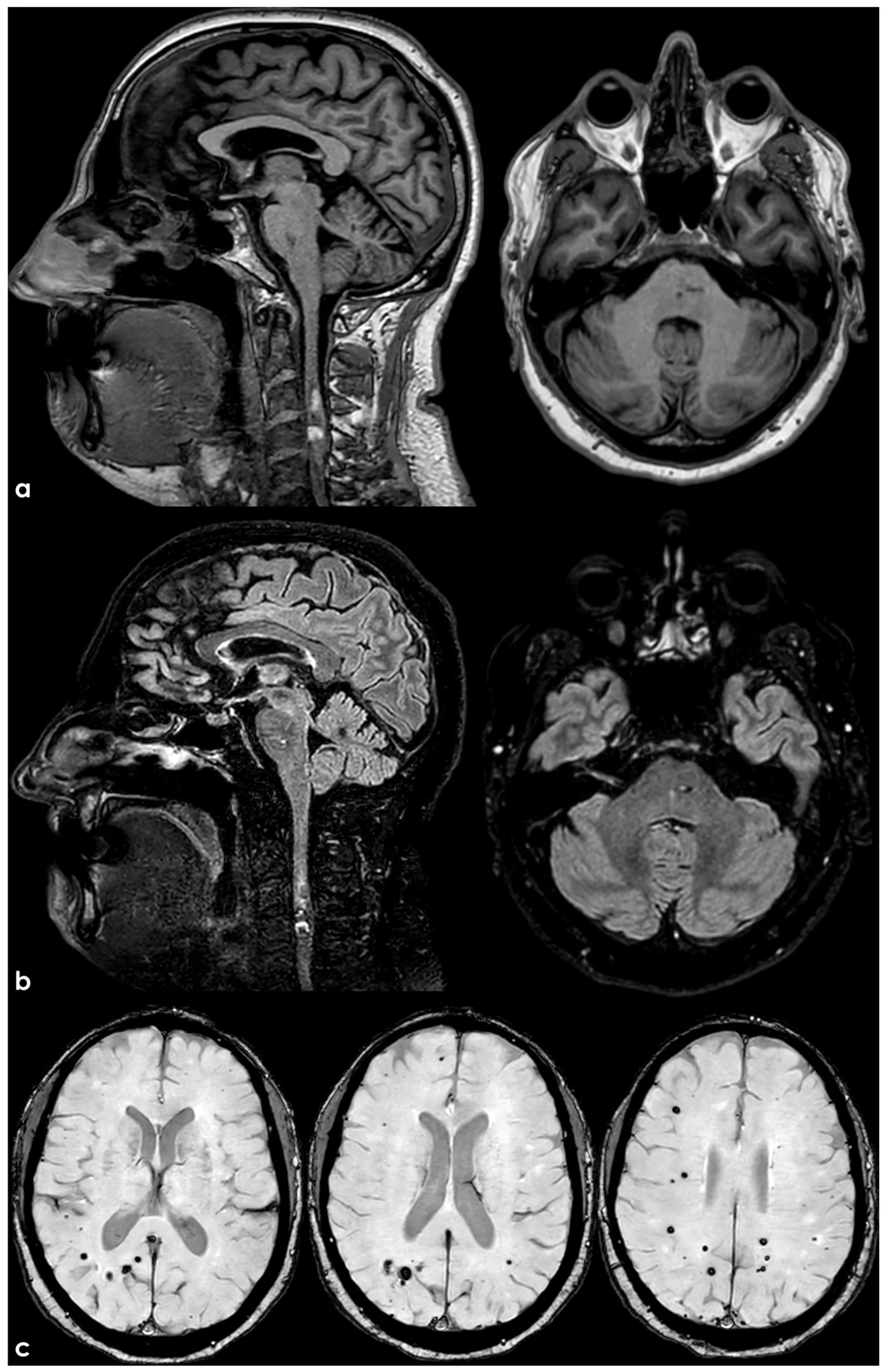
Brain MRI of a young patient complaining the subacute occurrence of paresthesias on the left side with head sparing. The MRI was performed at 3 weeks from symptoms onset. (**Panel a**): 3D T1W sequence reconstructed in sagittal (**left**) and axial plane (**right**), showing a left hemipontine hypodense lesion and two cervical tumefactive hyperintense intra-axial lesions. (**Panel b**): Same findings on sagittal (**left**) and axial 3D T2-Fluid Attenuated Inversion Recovery (FLAIR) sequence, showing an hyperintense signal in the cervical spinal lesions. (**Panel c**): SWI in axial view showing multiple rounded hypointensities suggesting CCMs (Zabramski 1 and 4).

**Figure 2:**
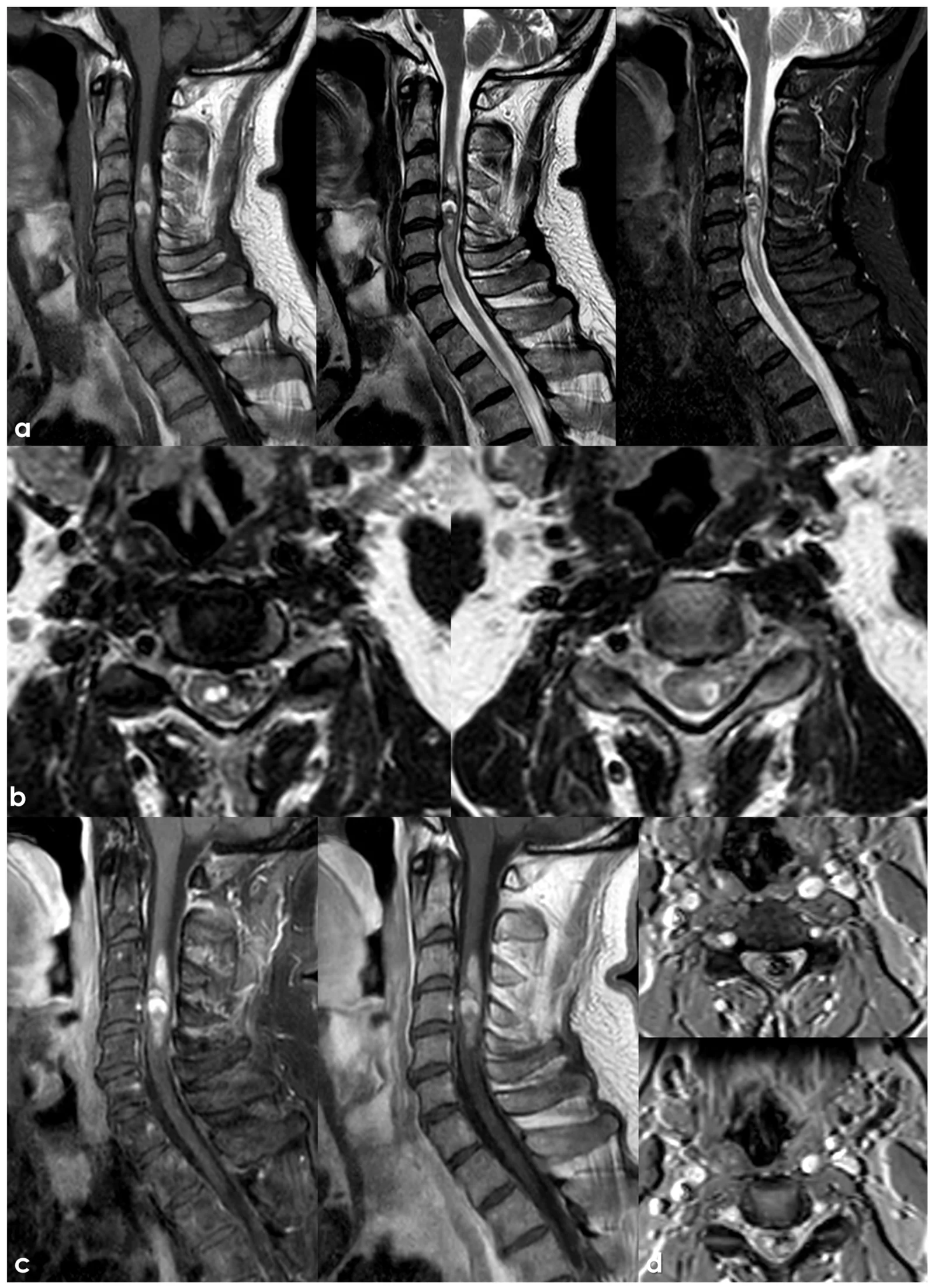
Cervical spine MRI in the same patient of Figure 1. (**Panel a**): Sagittal T1W, T2W and STIR sequence (from (**left**) to (**right**)), confirming a multiloculated rounded hyperintense lesion surrounded by mild edema with tumefactive effect. (**Panel b**): Axial T2W sequence showing at two contiguous levels the involvement of the lateral funiculus on the right hemicord and of the posterior funiculus on the left hemicord in a relatively deep position. (**Panels c**,**d**): Post-contrast T1 Dixon sequence in a sagittal and axial plane. All these findings are consistent for a subacute hemorrhage with an underlying intramedullary cervical cavernous angioma. The sister of the patient received a diagnosis of FCCM a few years before (frameshift mutation of *CCM2* gene) and, at that time, the patient refused to undergo genetic testing, although being highly susceptible to receiving the same diagnosis.

**Figure 3:**
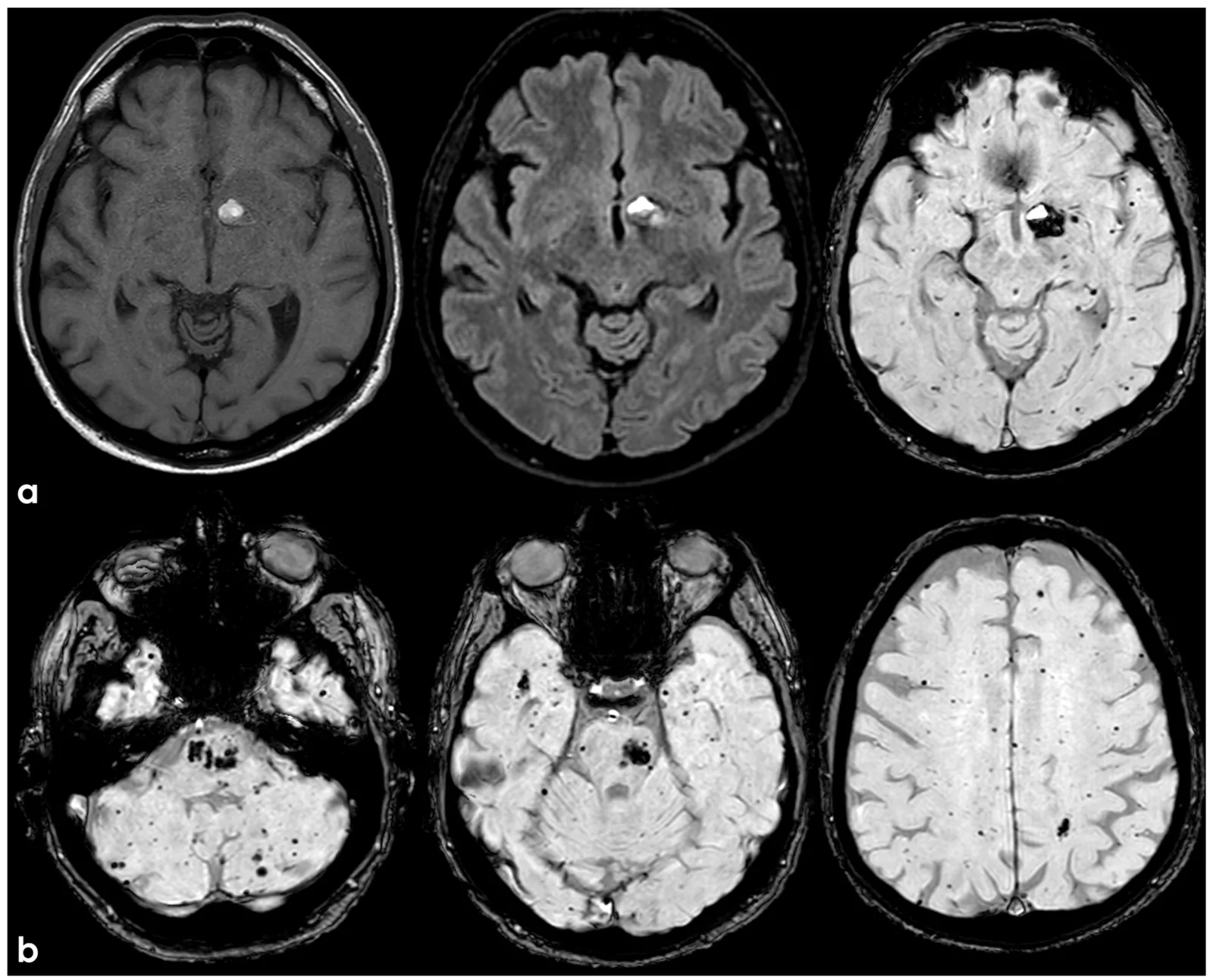
Brain MRI of a 74 yo man with a focal seizure. (**Panel a**): axial T2, FLAIR and SWI, showing a left deep anterior diencephalic lesion consistent with subacute bleeding of a cavernous angioma. (**Panel b**): Other axial SWI slices showing multiple hypointense lesions confirmed as CCMs. The patient underwent genetic testing, finding a known pathogenic mutation of *CCM1* gene.

**Figure 4:**
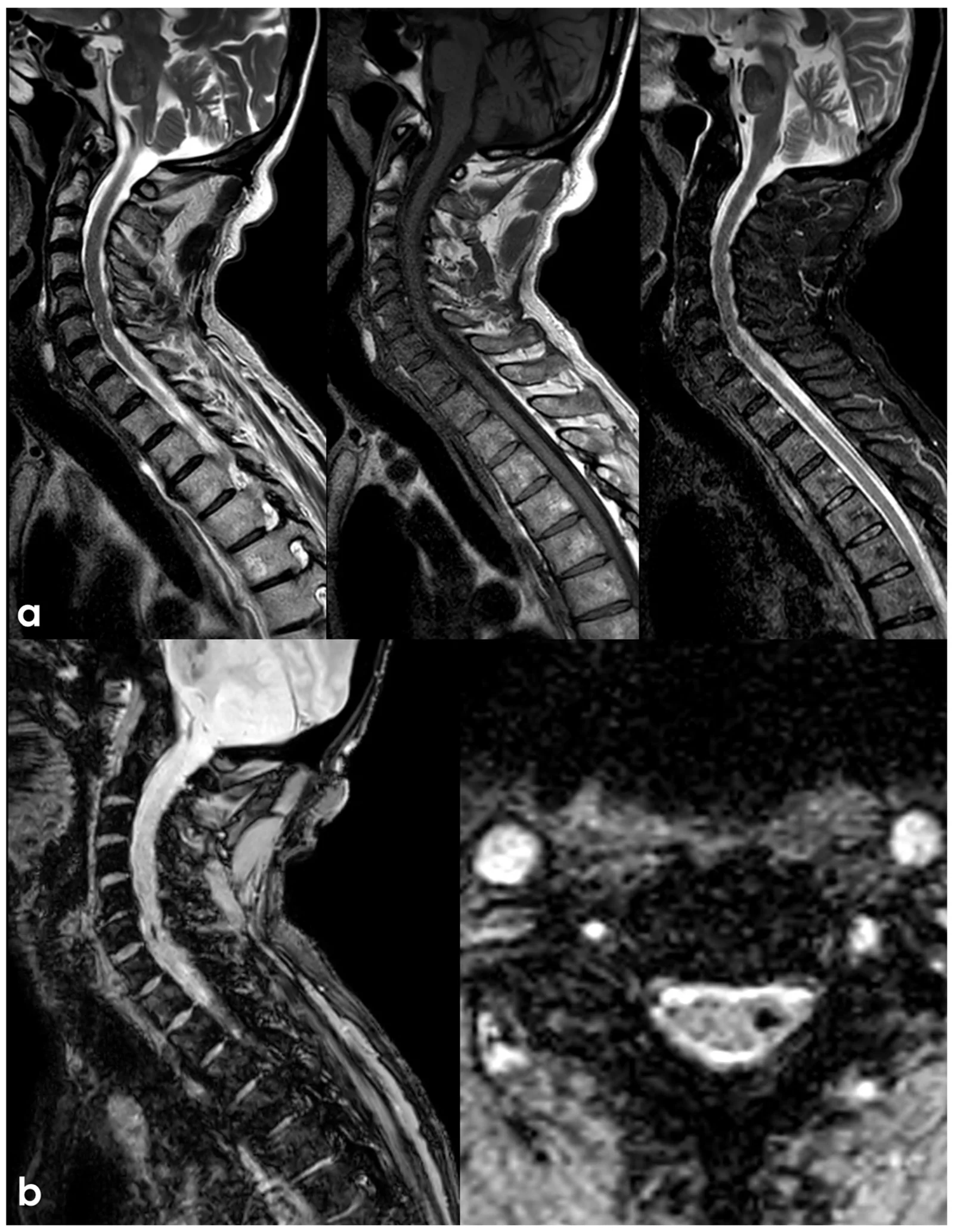
Cervical spinal MRI in the same patient of Figure 3, performed as screening for asymptomatic SCCMs. (**Panel a**): Sagittal T1W, T2W and STIR sequence (from (**left**) to (**right**)), showing a small rounded intramedullary lesion, with a dotted Y1W hyperintensity. (**Panel b**): GRE sequence in sagittal (**left**) and axial (**right**) acquisition, showing a correspondent hypointensity on the left lateral funiculus, consistent for SCCM.

**Figure 5:**
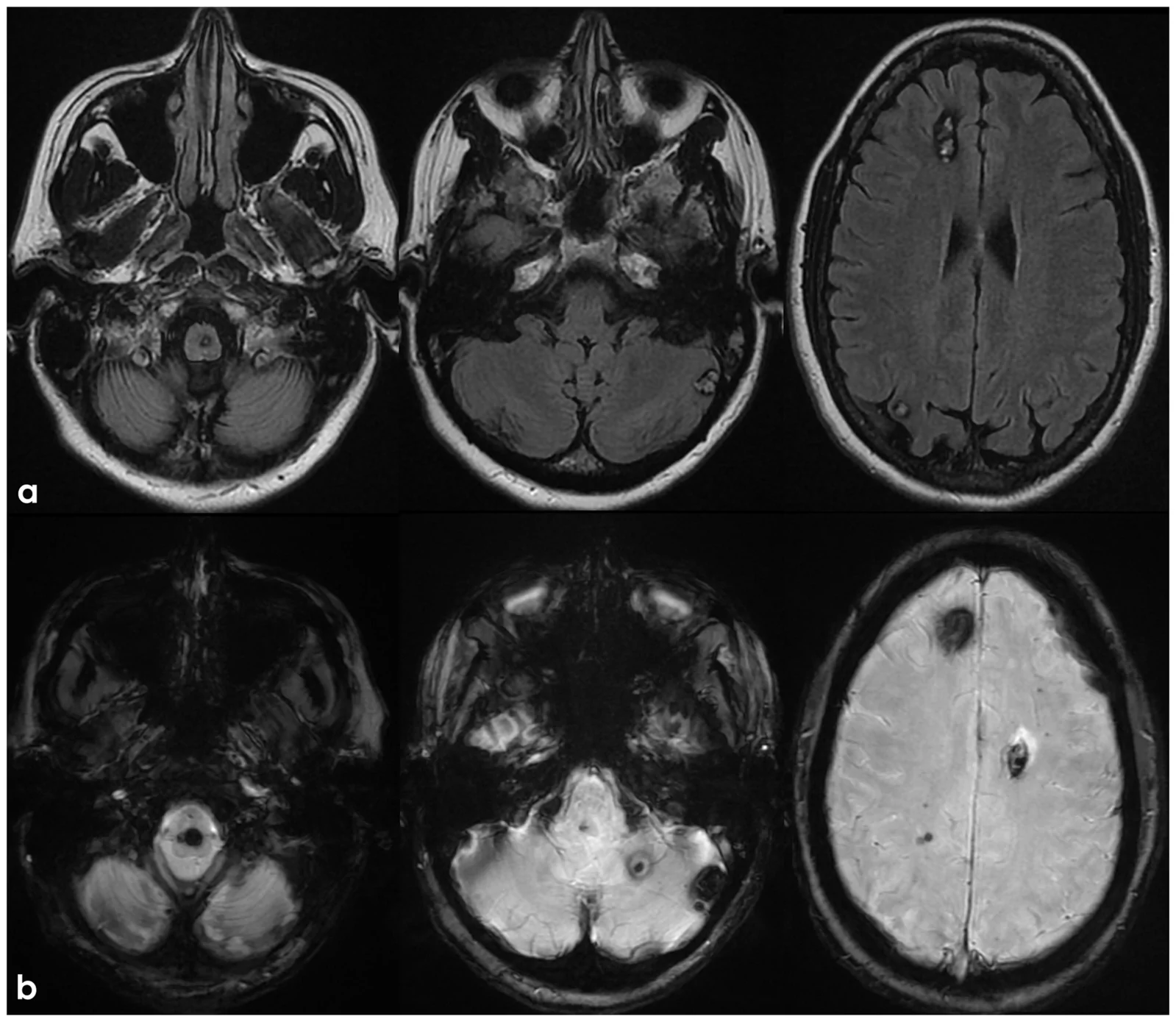
Brain MRI in a patient with a known FCCM (CCM2 gene mutation), diagnosed at 23 years of age due to the acute hemorrhage on a left frontal CCM. (**Panel a**): Axial FLAIR with the classic appearance of at least five CCMs. (**Panel b**): Axial SWI showing the correspondent hypointense signal.

**Figure 6:**
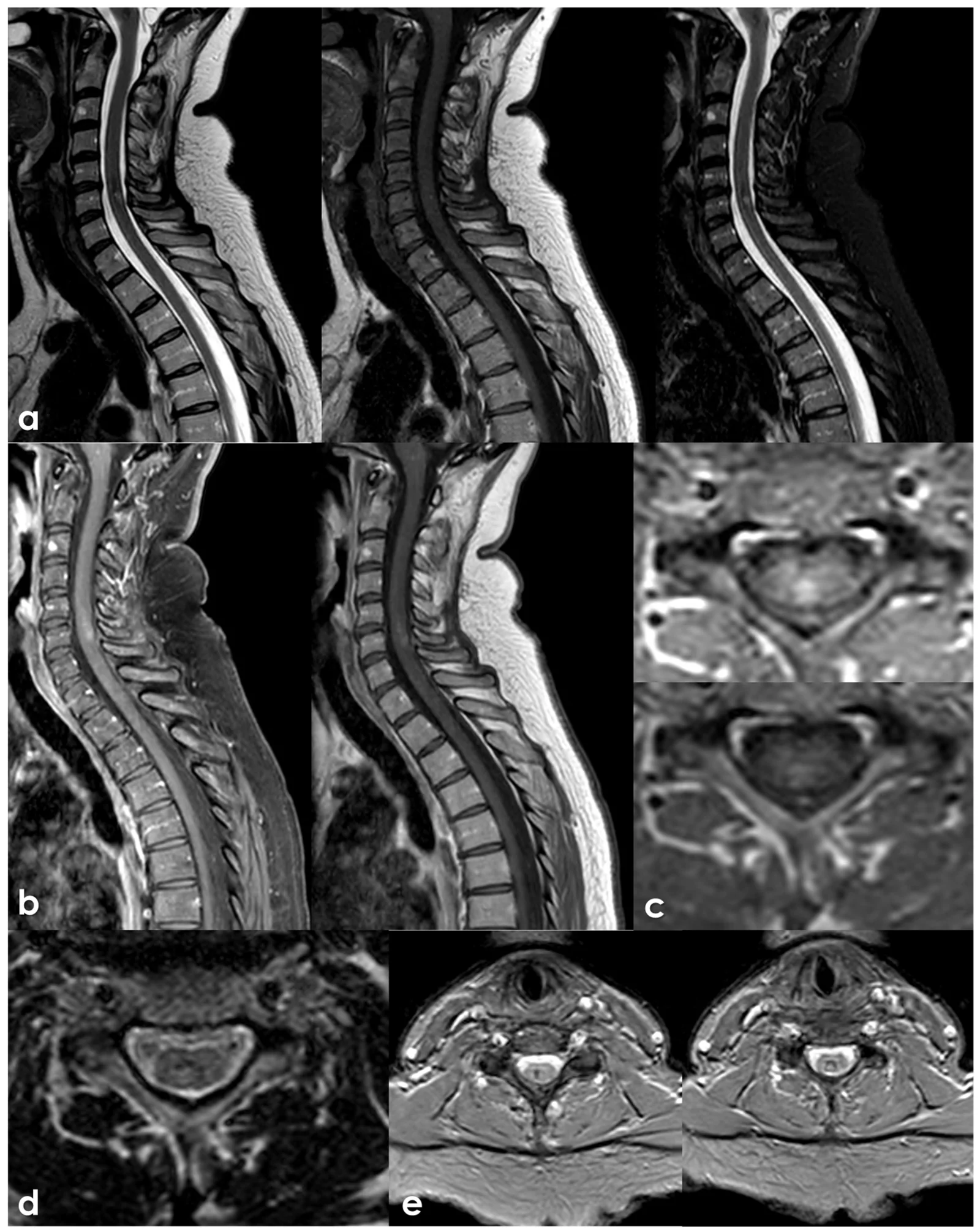
Spinal cord MRI in the same patient of Figure 5, performed about 45 days after the onset of ataxic gait and neurological pattern of posterior funiculus involvement with a cervical level. (**Panel a**): Sagittal T1W, T2W and STIR sequence (from (**left**) to (**right**)), showing a mildly T1-hyperintense lesion in the posterior half of the cervical spinal cord. (**Panel b**): Same lesion in sagittal post-gadolinium T1 Dixon sequence, showing intralesional contrast enhancement with a homogeneous pattern and smooth lining. (**Panel c**): Same appearance of panel b in axial plane, confirming the posterior median-paramedian location of the lesion. (**Panel d**): Axial T2W image showing a slightly hyperintense lesion with a hypointense border in the posterior funiculus. (**Panel e**): Axial GRE sequence with a corresponding hypointense signal. The findings are consistent with a cervical intramedullary SCCM with late subacute bleeding. The patient had a known diagnosis of FCCM due to a frameshift mutation of CCM2 gene, but spinal cord was not screened as potential site for cavernous malformation at the time of diagnosis. About 20 years after the diagnosis of FCCM, the patient developed a subacute myelopathy, leading to the identification if spinal bleeding due to an underlying cavernous angioma.

Some examples are provided in Figure 1, Figure 2, Figure 3, Figure 4, Figure 5 and Figure 6.

## 6. Management

The spinal location of CCMs is globally rare, and their prevalence may be underreported due to the lack of systematic studies focusing on the spinal cord in patients with brain CCMs [139]. This applies to both sporadic CCMs and FCCMs, where new lesions can emerge over a patient’s lifetime as de novo formations [140]. Consequently, research on spinal malformations is relatively limited compared to studies on brain CCMs. The Angioma Alliance guidelines do not provide specific recommendations for managing SCCMs, suggesting instead that they be treated similarly to brainstem cavernous malformations (BSCMs), considering the spinal cord’s high eloquence [138].

Reviews typically endorse a conservative management strategy for asymptomatic spinal CCMs, reserving surgical intervention for patients who experience a hemorrhagic event [78,115,141]. This conservative approach is warranted due to the risks associated with recurrent bleeding and the potential for impaired neurological recovery after subsequent hemorrhagic episodes [140]. Surgical decisions become particularly challenging for lesions that are hard to access, such as ventrally located non-exophytic CCMs or those situated in the thoracic or lumbar spinal cord. In such cases, surgery may be postponed until after a second hemorrhagic event, especially if the patient only presents mild neurological deficits [28,142].

Resection of intramedullary SCCMs should ideally occur at the most superficial point, often requiring varying degrees of bony removal. Sectioning the dentate ligament at multiple levels can aid in rotating the spinal cord during surgery for anterolateral malformations. For deeper lesions, the posterior median sulcus approach, guided by neuromonitoring, is the most commonly used technique. The posterolateral sulcus approach, located just posterior to the dorsal root entry zone, is beneficial for lateralized malformations [28,90]. While there is no expert consensus specifically addressing spinal CCMs, there is alignment with the guidelines for BSCMs. Most studies advocate that surgical intervention should occur within three months following a hemorrhage [140,142]. One of the few prospective studies in this area advises against early intervention, recommending that surgery be delayed until a dissection membrane has formed, akin to the approach for BSCMs [143].

A recent meta-analysis examined the safety and efficacy of conservative versus surgical management in patients with intramedullary SCCMs [144]. This analysis included 515 patients, of whom 343 (66.6%) underwent surgical management, while 172 (33.4%) received conservative treatment. Key findings include:

- Long-term Functional Outcomes: Surgical management resulted in significantly improved long-term functional outcomes (odds ratio (OR) 3.27, 95% confidence interval (CI) 1.72–6.24).
- Risk of Clinical Deterioration: There was no increase in the risk of clinical deterioration (OR 1.03, 95% CI 0.35–3.03).
- Hemorrhage Risk: Surgical management reduced the risk of hemorrhage during follow-up (1.69% vs. 17.3%).

This meta-analysis underscores the importance of evaluating treatment options and outcomes for SCCMs, indicating the potential benefits of surgical intervention even in complex cases. However, several limitations were identified in this meta-analysis:

(1) Selection Bias: Higher rates of surgery reported could lead to an overestimation of the benefits associated with surgical intervention.
(2) Heterogeneity: Variability in outcome measures and study designs complicates comparisons.
(3) Small Sample Size: The limited number of patients in some studies reduces the generalizability of the findings.
(4) Lack of Granularity: Insufficient data on specific variables, such as lesion size and depth, hampers detailed analysis.
(5) Retrospective Nature: Outcomes may be influenced by unmeasured variables, including surgeon expertise and institutional protocols.

Additionally, a recent systematic review [145] examined optimal treatment strategies for SCCMs, analyzing data from 2328 patients across 50 studies. The majority of these studies were retrospective and single-center, with a high risk of bias in 80% of the cases. Key findings from this review are summarized in Table 2.

Surgical treatment generally resulted in better neurological outcomes compared to conservative management, but the baseline characteristics of patients differed significantly between treatment groups, leading to potential biases in outcome interpretation. The authors call for more rigorous, prospective, multicenter studies to better inform treatment strategies. The findings highlight the complexity of treatment decisions for SCM due to the rarity of the condition, varying treatment practices, and the high risk of bias in existing studies. Future research is needed to establish clearer guidelines and to evaluate the long-term outcomes of both treatment strategies.

According to a previous meta-analysis, the factors associated with favorable outcomes of surgery are illustrated in Table 3 [78]. Moreover, the study found no significant correlation between patient age, sex, spinal level of lesion involvement, or lesion size with outcomes. Patients receiving conservative management exhibited poorer outcomes compared to those who underwent surgery.

These findings emphasize the importance of early intervention, complete resection, and the surgical approach in determining favorable neurological outcomes for patients with ISCCMs. These factors should be considered when devising treatment plans for affected individuals (Table 3).

## 7. Discussion

What emerges from the literature review is the extremely limited availability of information on the prevalence, characteristics, natural history, and prognosis of SCCMs in patients with FCCM. This is largely due to the fact that the vast majority of SCCM cases derive from surgical series on symptomatic patients and have not focused on etiological factors, thus systematic genetic screening has never been performed in these patients. On the other hand, patients with “sporadic” SCCMs diagnosed with FCCM have never been systematically screened for possible asymptomatic localizations of cavernous malformations in the spinal cord. Therefore, information is lacking on both fronts, and what is currently proposed derives from the extension of what is known about “sporadic” SCCMs to familial ones.

In fact, SCCMs can be asymptomatic, often discovered incidentally during imaging for unrelated issues. However, symptomatic cases typically present with neurological deficits correlated to the lesion’s anatomical location. Common presentations include motor deficits (60.5%), sensory deficits (57.8%), and pain (33.8%) [15]. The majority of SCCMs are located in the thoracic region, followed by the cervical area and the cervico-medullary junction [15]. Genetic studies have identified mutations in the *KRIT1*, *CCM2*, and *PDCD10* genes as significant contributors to the pathogenesis of FCCM, reinforcing the importance of genetic screening in these patients, but no association has been demonstrated between individual genes mutations and SCCMs as predisposing factor. The lack of systematic studies specifically targeting the spinal cord in patients with FCCM has led to underreporting of SCCMs and their associated pathogenesis and epidemiology.

MRI is the gold standard for diagnosing SCCMs, as these lesions are angiographically occult; however, the sensitivity and specificity of MRI cannot be assessed due to the lack of a valid gold standard as comparator, with MRI inn this case being the only gold standard in a pathological study. The recommended imaging techniques include T1- and T2-weighted sequences, T2 FLAIR, and SWI as extensions of brain to spinal cord examinations. While MRI can effectively identify and characterize SCCMs, the absence of a standardized classification for spinal lesions limits the ability to predict hemorrhage risk based on imaging alone. The Zabramski classification, which categorizes CCMs based on imaging and histological characteristics, has not yet been validated for SCCMs. A modified classification has been proposed, distinguishing between types of lesions based on their signal intensity on MRI, which may aid in predicting clinical outcomes.

Current management strategies for SCCMs emphasize conservative treatment for asymptomatic cases, reserving surgical intervention for patients who have experienced hemorrhagic events. Surgical resection remains the primary treatment for symptomatic patients, particularly given the implications of recurrent bleeding on neurological recovery. Surgical challenges are pronounced for deeper lesions, often necessitating advanced techniques and careful planning to minimize complications [15]. Guidelines recommend surgery within three months of hemorrhagic events to improve outcomes, although some studies suggest delaying surgery until a dissection membrane forms could be beneficial.

Despite advances in understanding SCCMs and their potential association with FCCM, significant gaps remain in the current literature. The rarity of these lesions means that data is primarily drawn from retrospective studies, which often suffer from selection bias. Most findings pertain to surgical cases, leading to underrepresentation of asymptomatic patients and potential overestimation of surgical benefits. Furthermore, there is a lack of consensus on the best approaches for screening SCCMs in patients with FCCM or on the implications of identifying SCCMs in patients with brain CCMs.

Future research should focus on several key areas to address existing knowledge gaps:

- Systematic screening protocols: Lacking guidelines for routine spinal imaging in patients diagnosed with FCCM, a dedicated consensus could be proposed to collect information and better understand the prevalence and natural history of SCCMs in patients with FCCM.
- Systematic genetic testing: A systematic genetic screening in patients with SCCMs could be useful to identify different phenotypes and natural history in sporadic and familiar SCCMs.
- Longitudinal studies: Conducting prospective, multicenter studies to evaluate the natural history of SCCMs, including the development of new lesions and the impact of genetic factors on disease progression, may provide the basis for testing the pharmacological therapies considered in CCMs on SCCMs.
- Molecular mechanisms: Investigating the molecular pathways involved in the formation of SCCMs, particularly in the context of identified genetic mutations, to inform targeted therapeutic strategies.
- Standardizing classification: Developing and validating a standardized classification system for SCCMs that could assist in predicting clinical outcomes and guiding treatment decisions.

## 8. Conclusions

SCCMs associated with FCCM present distinct challenges in terms of diagnosis and management. There is a pressing need for increased awareness among clinicians regarding the hereditary nature of these lesions and the importance of comprehensive screening. By addressing the gaps in knowledge and establishing clear research directions, we can improve clinical outcomes for individuals affected by this condition.

## Figures and Tables

**Table 1:** Pathogenesis and pathophysiology of FCCM.

**Overview**	- FCCM is a heritable disease characterized by brain and spinal CCMs. - Mutations in the involved genes affect endothelial junction biology, mechanotransduction, and kinase signaling, leading to vascular instability.
**Genetic Mechanism**	- FCCM follows a “two-hit” mechanism: Inheritance of a germline loss-of-function (LOF) mutation in one allele of a CCM gene. A somatic second hit that inactivates the remaining wild-type allele in endothelial cells. - Major genes involved include: ***KRIT1/CCM1*** (53–65% of cases); ***CCM2/MGC4607*** (10–16%); ***PDCD10/CCM3*** (5–15%).
**Development of Lesions**	- Lesions develop from clonal expansion of CCM-deficient endothelial cells. - Mosaic mouse models show that a small number of mutant endothelial cells can seed and expand a lesion, recruiting surrounding wild-type cells.
**Endothelial Dysfunction**	- All CCMs exhibit disorganization of interendothelial junctions, leading to chronic leakage and deposits of blood degradation products. - Histopathological studies confirm active angiogenesis and inflammatory cell infiltration, indicating ongoing biological activity.
**Cell Adhesion and Signaling Pathways**	- CCM proteins (*CCM1, CCM2, CCM3*) cooperate to stabilize endothelial junctions and regulate signaling networks: RhoA/ROCK pathway hyperactivation is critical in the cavernous phenotype. Disruption of adhesion complexes involving VE-cadherin leads to increased RhoA signaling, actomyosin contractility, and loss of cell–cell adhesion.
**Kinase Activity Dysregulation**	- Dysregulated kinase activity is a hallmark of CCM progression: The **MEKK3–MEK5–ERK5–KLF2/4** pathway is upregulated in CCM-deficient endothelial cells, promoting RhoA activation. Receptor tyrosine kinase (RTK) signaling is also affected, contributing to abnormal angiogenic signaling.
**Immunoinflammatory Contributions**	- Inflammatory pathways and genetic modifiers play roles in lesion burden. - Endothelial Toll-like receptor 4 (TLR4) signaling is implicated in lesion formation, influenced by microbiome-derived signals.
**Vitamin D and Other Modifiers**	- Low vitamin D levels are associated with aggressive disease behavior, while supplementation may reduce hemorrhage risk.

**Table 2:** Main issues in treatment strategies of SCCMs [145].

Issues	Findings
Demographics and Treatment	Mean age of patients was 42 years, with 47% being women. Surgical treatment was performed in 82% of cases, while 18% received conservative management.
Neurological Outcomes	Surgical Treatment: 45% of patients showed improvement in neurological status post-surgery. 72% were able to ambulate independently after surgery, up from 60% preoperatively. Conservative Management: Only 16% showed improvement in neurological status. 76% were ambulating independently at follow-up, slightly down from 79% at baseline.
Hemorrhage Rates	The pooled annual hemorrhage rate was 4.8% during follow-up. Higher rates were observed in studies with shorter follow-up durations.
Risk of Bias	The majority of studies had serious risks of bias due to confounding factors, complicating direct comparisons between treatment outcomes

**Table 3:** Factors associated with a favorable outcome of surgical management [78].

Issue	Features
Timing of surgery	Patients who undergo resection within 3 months of symptom onset show significantly better outcomes (OR 2.11).
Extent of resection	Gross-Total Resection: Achieving complete removal of the cavernous malformation is associated with improved outcomes (OR 3.61).
Surgical approach	Hemilaminectomy is a minimally invasive approach linked to better outcomes compared to total laminectomy or laminoplasty (OR 3.20).
Clinical course	Patients with an acute, stepwise deterioration of symptoms have better outcomes (OR 1.72).
Presenting symptoms	Motor Symptoms: Presence of motor symptoms correlates with favorable outcomes (OR 1.76). Sensory Symptoms: Conversely, sensory symptoms are associated with poorer recovery (OR 0.58).
Duration of symptoms	A clinical history of 5 years or less is linked to better outcomes in conservative management (OR 10.0).

## Data Availability

No new data were created or analyzed in this study. Data sharing is not applicable to this article.
